# Determining *spa*-type of methicillin-resistant *Staphylococcus aureus* (MRSA) via high-resolution melting (HRM) analysis, Shiraz, Iran

**DOI:** 10.1186/s13104-020-04948-z

**Published:** 2020-02-24

**Authors:** Zahra Hashemizadeh, Abdollah Bazargani, Davood Kalantar-Neyestanaki, Samane Mohebi, Nahal Hadi

**Affiliations:** 1 Department of Bacteriology and Virology, School of Medicine, Shiraz University of Medical Science, Shiraz, Iran; 2 Medical Mycology and Bacteriology Research Center, Kerman University of Medical Sciences, Kerman, Iran; 3 Department of Microbiology and Virology, School of Medicine, Kerman University of Medical Sciences, Kerman, Iran; 4 Bioinformatics and Computational Biology Research Center, Shiraz University of Medical Sciences, Shiraz, Iran

**Keywords:** MRSA, HRM, *spa* type

## Abstract

**Objectives:**

Molecular typing methods are useful for rapid detection and control of a disease. Recently, the use of high-resolution melting (HRM) for *spa* typing of MRSA isolates were reported. This technique is rapid, inexpensive and simple for genotyping and mutation screening in DNA sequence. The aim of this study was to evaluate the ability of HRM-PCR to analysis *spa* genes amongst MRSA isolates.

**Results:**

A total of 50 MRSA isolates were collected from two teaching hospitals in Shiraz, Iran. The isolates were confirmed as MRSA by susceptibility to cefoxitin and detection of *mecA* gene using PCR. We used HRM analysis and PCR-sequencing method for *spa* typing of MRSA isolates. In total, 15 different *spa* types were discriminate by HRM and sequencing method. The melting temperature of the 15 *spa* types, using HRM genotyping were between 82.16 and 85.66 °C. The rate of GC % content was 39.4–46.3. According to the results, *spa* typing of 50 clinical isolates via PCR-sequencing and HRM methods were 100% similar. Consequently, HRM method can easily identify and rapidly differentiate alleles of *spa* genes. This method is faster, less laborious and more suitable for high sample at lower cost and risk of contamination.

## Introduction

MRSA is one of the most common nosocomial pathogens worldwide, leading to severe morbidity and mortality. In *Staphylococcus aureus*, the *mecA* gene causes resistance to methicillin. This gene produces PBP2A (protein binding to penicillin) which inhibits beta-lactam antibiotics [1]. Molecular typing methods are useful for rapid identification and to control disease [2]. Nowadays, pulsed-field gel electrophoresis (PFGE) is the gold standard method for the typing of *S. aureus* isolates; however, this method is time consuming process, expensive, complicated, and difficult to standardize [3]. Amongst molecular typing methods, SCC*mec* typing method is simple and cost effective with high discriminatory power to detect MRSA isolates associated with nosocomial infections [3]. In recent years, molecular typing of *S. aureus* has been performed by *spa* sequence typing [4–6]. The Staphylococcus protein A (*spa*) gene has the polymorphic in X region, which determines by sequencing method [5, 6].

In recent years, high-resolution melting (HRM) analysis has been used to analyze genetic variations. This technique is rapid, inexpensive, simple and cost effective for genotyping and mutation screening in DNA sequence [7]. HRM with real-time polymerase chain reaction (PCR) can be used for *spa* typing hypervariable X region of the *spa* gene [7–11]. In this technique, DNA strands are distinguished based on the length and percentage of the GC content [11]. The aim of this study was to assess the ability of HRM-PCR in analyzing *spa* genes amongst MRSA isolates.

## Main text

### Materials and methods

#### Bacterial isolates

In this study, from January 2017 to June 2018, a total of 50 MRSA isolates were collected from different patients. All isolates were identified as MRSA by standard biochemical tests, resistance to cefoxitin and detection of *mecA* gene, as it was described previously [12].

#### Spa typing

The X region of the *spa* gene was amplified, using spa-1113f (5-TAA AGA CGA TCC TTC GGT GAG C-3) and spa-1514r (5-CAG CAG TAGTGC CGT TTG CTT-3) primers [5]. The PCR assay contained the following components per reaction: 12.5 µL Master mix (Amplicon, Denmark), 0.2 µL of each primer with concentration of 10 pmol/µL and 2 µL of DNA template top up to 25 µL. PCR products were sequenced (Macrogen, Co, Korea) and assigned according to (http://www.spaserver.ridom.de).

#### High-resolution melting PCR method

The genomic DNA was extracted using Exgene Clinic SV (GeneALL, Seoul, Korea) according to manufacturer’s guidelines and primer pair spa (spa-1113f and spa-1514r) were applied to the HRM method.

The HRM analysis was performed by the Quant studio real-time PCR system (ABI bio system, USA). HRM was performed in 25 µL reaction containing 5 µL of 5 HOT FIREPol EvaGreen HRM Mix (ROX) (Solis BioDyne Co, Estonia), 1 µL of each primer (10 pM), 2 µL of DNA template (20 ng) and 16 µL of water (DNase and RNase free water, SinaClon BioScience Co, Iran). The PCR thermocycling condition was: 95 °C for 12 min, 40 cycles at 95 °C for 15 s, 63 °C for 20 s and 72 °C for 20 s. Then, 95 °C for 1 min and 40 °C for 1 min followed by HRM ramping from 75 to 90 °C with fluorescence data acquisition at 0.5 °C increments. The melting curves of all samples were analyzed by HRM Software v3.0 (Thermo Fisher Scientific Co, USA).

### Results

In the present study, total of 50 MRSA isolates were analyzed. Overall, 26 and 24 MRSA isolates were collected from female and male patients. The study group age range was between 8–90 years. The MRSA isolates were obtained from patient’s skin (19, 38%), Blood (10, 20%), wound (8, 16%), Sputum (5, 10%), nasal (5, 10%), fluid (1, 2%), abases (1, 2%) and eye (1, 2%). Samples were isolated from different wards including Dermatology (17, 34%), Internal (7, 14%), Emergency (7, 14%), ICU (6, 12%) and outpatient (12, 24%).

Using conventional PCR-sequencing analysis for the 50 clinical isolates of MRSA, showed 15 different *spa* types, including *spa* types; t021, t018, t030, t037, t386, t081, t325, t345, t790, t314, t186, t304, t003, t1877, t1816. *Spa* type t030 was the major type in this study. These *spa* types were defined as the control for HRM analysis. The processing time for HRM analysis was around 2 h. The melting temperature of the 15 HRM genotypes was between 82.16 and 85.66° C. The rate of GC % lies between 39.4 and 46.3 (Table 1). Typing of the 50 clinical isolates by the PCR-sequencing method and HRM method resulted in 100% similarity (Table 1).

**Table 1:** *Spa* typing results of the 50 MRSA clinical isolates, using conventional PCR-sequencing method and HRM

*Spa* types	No. of isolates (n:50)	PCR base spa types	Tm (°C)	GC %	Size bp
t 030	7	15-12-16-02-24-24	83.83	44	250
t 037	2	15-12-16-02-25-17-24	82.16	44.6	360
t 021	5	15-12-16-02-16-02-25-17-24	85.3	43.9	300
t 386	4	07-23-13	82.96	42.2	210
t 325	2	07-12-21-17-34-13-34-34-33-34	82.95	41.1	400
t 345	1	26-23-13-21-17-34-34-33-34	85.18	41.7	350
t 790	1	26-23-13-23-31-29-17-25-17-25-16-28	84.13	44.1	450
t 314	1	08-17-23-18-17	85.66	44.6	270
t 186	1	07-12-21-17-13-13-34-34-33-34	84.5	41.8	300
t 304	1	11-10-21-17-34-24-34-22-25	84.35	42.8	300
t 003	1	26-17-20-17-12-17-17-16	85.33	44.4	280
t 1877	3	07-23-12-34-12-12-23	84.38	44.4	290
t 1816	1	07-12-21-17-34-13-34-34-34-33-34	84.57	39.4	400
t 018	1	15-12-16-02-16-02-25-17-24-24-24	85.1	44.8	264
t 081	1	04-21-12-41-20-17-12-17	85.3	46.3	192

### Discussion

MRSA can cause infections both in hospitals as well as communities [13, 14]. Due to outbreaks of MRSA increase in the mentioned settings, typing methods are vital to systematically control these infections. Molecular typing methods of microbial pathogens are important tools for detecting the outbreaks, controlling and preventing infections as well as for the epidemiological surveillance [5]. Different typing methods are done for epidemiological investigations such as PFGE, multilocus sequence typing (MLST), *spa* typing and SCC*mec* typing [14]. PFGE and MLST are appropriate typing methods; however, these methods are expensive and time-consuming [15]. *Spa* typing method is based on short sequence repeats of hypervariable X region in the *spa* gene, but this method is still expensive. But then again this typing method is effective and easy technique to detect typing MRSA isolates [16]. In a previous study, we found 15 different *spa*- types, and spa type t030 was the most common amongst MRSAs [17]. HRM analysis has been recently introduced as an appropriate method for epidemiological investigations and detection of sequence variants in clinical research and diagnostics. HRM method can easily and rapidly identify the different alleles of *spa* genes [11]. This method is used in a closed system and in the same tube as the amplification step; hence, it is faster, easier, and more suitable for high sample, which reduces the risk of contamination [18]. In our study, the results of HRM showed 15 different *spa* types. All of the isolates had significant difference in Tm and GC content. The G + C percentage and the length of the tandem repeats in the X region of *spa* gene might cause differences in melting temperature among the samples. Our *spa* typing results was completely similar to HRM results. This finding is in line with the findings of Hon-Kwan Chen et al. study in China [8]. However, our results were in contrast with the results of Fasihi et al. [11] and Stephens et al. [10], which showed that for some isolates, Tm HRM with conventional PCR sequencing were different. HRM is a suitable method to be used to examine hypervariable loci, since it can provide more clearance for genotyping and diversity among MRSA strains. Also, another study claimed that this technique is less expensive than *spa* sequencing [19]. *Spa* typing using with other typing methods, such as MLST and SCC*mec* can be distinguished better amongst MRSA clones in hospital [19]. Different values of melting temperature were reported for one *spa* type. HRM method can be affected by testing conditions and type of real time machine as well as HRM software [9, 11]. In this study, Tm value for t030 was 83.83 °C, but in a previous study by fasihi et al. it was 85.3 °C. Also, in the present study as well as a previous study by Fasihi et al. HOT FIREPol EvaGreen HRM Mix (ROX) was used, but in another study Platinum SYBRGreen was used [9]. In our study, using HRM technique, we used Quant studio real-time PCR system (ABI bio system, USA) and 5 × HOT FIREPOL EvaGreen HRM Mix (ROX) (Solis Bio Dyne Co, Estonia) while in another study, a different real time machine and master mix was used [8, 9]. These differences can be as result of using different real time PCR device or Master Mix producing different TMs for the same *spa* type. Even though HRM method is simple, and more economical than the conventional method, it has several limitations. The HRM method cannot be replaced by sequencing and it requires the presence of positive controls for each polymorphism. In this technique it is essential to have a known control type; however, if there is a new *spa* type conventional PCR-sequencing is required. Also, isolates that have the same GC content with the same TM, their discrimination is difficult [8]. In HRM the basis of differentiation is fragment length, sequence, and GC percentage. So, as mentioned above, the greater the difference, it will be possible to separate and differentiate is greater too and vice versa.

### Conclusion

HRM method is simple, more economical than the conventional method. HRM method can easily and rapidly identify the different alleles of *spa* genes. This method can analysis faster is less laborious, more suitable for high quantity samples with lowers risk of contamination. Accordingly, this technique has the potential to show variation, but require control, and should be accompanied by the *spa* gene sequence, and it is not suitable for new strains.

## Limitations

A limitation in this study was that we did not carry out HRM for MSSA isolates, due to financial constraints.

## Data Availability

The data that support the findings of this study are available. Anyone interested can get upon reasonable request from corresponding author.

## Abbreviations

MRSA: Methicillin-resistant *S. aureus*
HRM: High-resolution melting
SCC*mec*: Staphylococcal cassette chromosome mec
MLST: PBP2A (protein binding to penicillin multilocus sequence typing)
PFGE: Pulsed field gel electrophoresis
CLSI: Clinical and Laboratory Standard Institute
